# Impact of malnutrition and protein-enriched oral nutrition on early recovery after fragility fractures in older adults: the NuTra trial

**DOI:** 10.1186/s12877-026-08247-3

**Published:** 2026-10-17

**Authors:** Carolina Vogel, Moritz Heyl, Louis Vollmer, Julia Schuhmacher, Johanna Demissie, Dannik Haas, Benedikt J. Braun, Steven C. Herath, Tina Histing, Mika F. Rollmann

**Affiliations:** 1 University Hospital Tuebingen, Tuebingen, Germany; 2 Department of Trauma and Reconstructive Surgery, University of Tuebingen, BG Trauma Center Tuebingen, Tuebingen, Germany

**Keywords:** Malnutrition, Frailty, Geriatric trauma, Protein supplementation, Functional recovery, Discharge destination

## Abstract

**Background:**

Malnutrition is highly prevalent in geriatric trauma patients and is associated with impaired recovery, complications, and reduced likelihood of returning home. Nonetheless, the impact of a targeted protein-enriched diet during the acute hospital stay on patient outcome and rehabilitation remains to be investigated.

**Materials and methods:**

This single-center randomized controlled study enrolled patients ≥ 70 years with fragility fractures admitted to a Level I trauma center. Nutritional status was assessed using the Nutritional Risk Screening 2002 and Mini Nutritional Assessment. Patients screened positive in ≥ 1 tool were classified as being at nutritional risk and randomized to a protein-enriched diet (Group A) or standard diet (Group B); patients with negative screening served as observational reference group without nutritional risk (Group C). The intervention targeted 1.5–2.0 g/kg/day protein. Primary outcomes were in-hospital medical complications, surgical site infections and functional mobility at discharge. Secondary outcomes included length of stay, discharge destination, and in-hospital mortality. Multinomial logistic regression assessed independent associations with discharge destination.

**Results:**

Of the 218 patients (mean age 81.8 ± 6.9 years; 72.0% female), 100 (45.9%) screened positive for being at nutritional risk and were randomized to intervention (A, *n* = 46) or control (B, *n* = 54), while 118 (54.1%) patients without nutritional risk served as reference group (C). Patients at nutritional risk showed higher comorbidity burdens, higher ASA classes, and greater frailty than group C. Medical complications occurred in 45.0% of patients, with no significant differences between groups. Serum albumin decreased and CRP increased during hospitalization, without differences among the groups. Patients in reference group C were more often independently mobile and discharged home, whereas all patients at nutritional risk more frequently required institutional care. In multivariable analysis, a higher age-adjusted CCI was independently associated with discharge destination, whereas no independent effect of the nutritional intervention was observed.

**Conclusion:**

In this geriatric trauma cohort, being at nutritional risk was closely linked to frailty and multimorbidity. A protein-enriched diet during the acute hospital stay did not translate into measurable short-term benefits regarding complications, functional mobility, length of stay or discharge destination. Nutritional risk should therefore be considered within the broader context of overall vulnerability, and nutritional care may need to be embedded in comprehensive longitudinal rehabilitation strategies extending beyond the acute in-hospital phase.

**Trial registration:**

The study was retrospectively registered at ClinicalTrials.gov on 12 May 2026 (NCT07582575).

**Supplementary Information:**

The online version contains supplementary material available at https://doi.org/10.1186/s12877-026-08247-3.

## Background

The ageing of the global population is accompanied by a marked rise in fragility fractures. In orthopedic trauma surgery, an increasing proportion of admissions now involves older adults presenting with major fragility fractures of the proximal femur, pelvis, vertebral body or proximal humerus [1–5]. These injuries often represent turning points in the life of geriatric patients, not only leading to sustained loss of mobility and self-care capacity, reduced quality of life and excess mortality, but also impose a substantial burden on healthcare resources [6–8]. Optimal care for this vulnerable patient population requires an interdisciplinary orthogeriatric approach combining timely surgical fracture management with systematic assessment and treatment of comorbidities and geriatric syndromes to enable fracture healing, regain mobility and, ideally, return to the pre-fracture living situation [9].

Malnutrition is a major, yet often underestimated component of this orthogeriatric co-management [10–13]. Its prevalence in older adults with fragility fractures has been reported in the range of approximately 19–28%, with higher rates in chronically ill patients, women and individuals aged over 80 years [11, 12, 14, 15]. Malnutrition commonly coexists with frailty and sarcopenia and may therefore serve as a surrogate marker of overall vulnerability rather than an isolated condition [16].

From a biomechanical perspective, low body mass index and reduced soft-tissue envelope increase local stress at impact and are associated with an increased risk of fractures, particularly at the hip [12]. From a clinical perspective, malnutrition has been linked to impaired fracture healing, poorer functional recovery, higher rates of medical and surgical complications and increased mortality [12, 14, 17–19]. In hip fracture cohorts, patients with malnutrition or at risk of malnutrition exhibit activities of daily living disability, as reflected by lower Barthel Index scores [18].

Several professional societies now recommend systematic nutritional screening and targeted nutritional support for older inpatients, including those with fragility fractures. In particular, protein intake of at least 1.2–1.5 g/kg body weight per day, and up to 2.0 g/kg in the context of acute illness, is advised to mitigate catabolism, support wound and fracture healing and maintain muscle mass [10, 20]. Nevertheless, the evidence base for peritraumatic oral nutritional supplementation (ONS) in geriatric trauma remains heterogeneous. Randomized trials and meta-analyses in hip fracture patients suggest that ONS can improve certain outcomes, such as overall complication rates, pressure ulcer incidence and, in some studies, functional recovery, whereas effects on surgical site infections, length of hospital stay and mortality are less consistent [21–24]. Importantly, most interventional studies to date have focused on hip fractures and often implemented nutritional strategies that extended into the post-acute rehabilitation phase [12, 14, 18, 19, 21–23, 25, 26]. Data on broader geriatric trauma populations, including pelvic, vertebral body and upper extremity fragility fractures, are scarce and the clinical impact of a structured protein-enriched diet initiated during the acute hospital stay remains unclear.

Against this background, the Nutrition and Trauma (NuTra) study was designed as a prospective clinical trial to evaluate the association between nutritional risk and early clinical outcomes in older patients with fragility fractures and to investigate whether a protein-enriched hospital diet supplemented with ONS improves these outcomes in patients at nutritional risk. The primary outcomes were in-hospital complications and functional mobility at discharge; secondary outcomes included length of hospital stay, discharge destination, and mortality. We hypothesized that patients at nutritional risk would have less favorable early clinical outcomes than patients without nutritional risk and that, among patients at nutritional risk, those receiving the protein-enriched diet would experience fewer complications, better functional mobility at discharge, and a greater likelihood of returning home than those receiving the standard hospital diet.

## Methods

### Aim

The aim of this study was to evaluate whether a protein-enriched diet during acute hospitalization improves short-term outcomes in geriatric trauma patients at nutritional risk.

### Study design and setting

The Nutrition and Trauma (NuTra) study is a monocentric, prospective study comprising a randomized controlled interventional component and a parallel observational cohort, conducted from May 1, 2024 to June 1, 2025 at an academic Level I trauma center in Germany. Outcomes related to benefits and harms were assessed during the index hospitalization and followed until hospital discharge.

### Characteristics of participants and eligibility criteria

Patients aged ≥ 70 years who were admitted for acute management of a fracture clinically classified as a fragility fracture were screened for eligibility. Eligible fracture types included fractures of the proximal humerus, vertebral body, pelvis, proximal femur and periprosthetic fractures of the lower extremity. Classification as a fragility fracture was based on the clinical assessment and the predefined eligible fracture types. Neither a previous diagnosis of osteoporosis nor DXA-confirmed osteoporosis was required for inclusion. Trauma mechanisms and DXA findings were not systematically recorded in the study database and were therefore not used for fracture classification. Patients were required to be able to provide informed consent. Exclusion criteria were dementia or relevant cognitive impairment precluding informed consent, progressive malignancy, palliative treatment goals and refusal or inability to provide consent.

### Nutritional assessment and group allocation

**Fig. 1:**
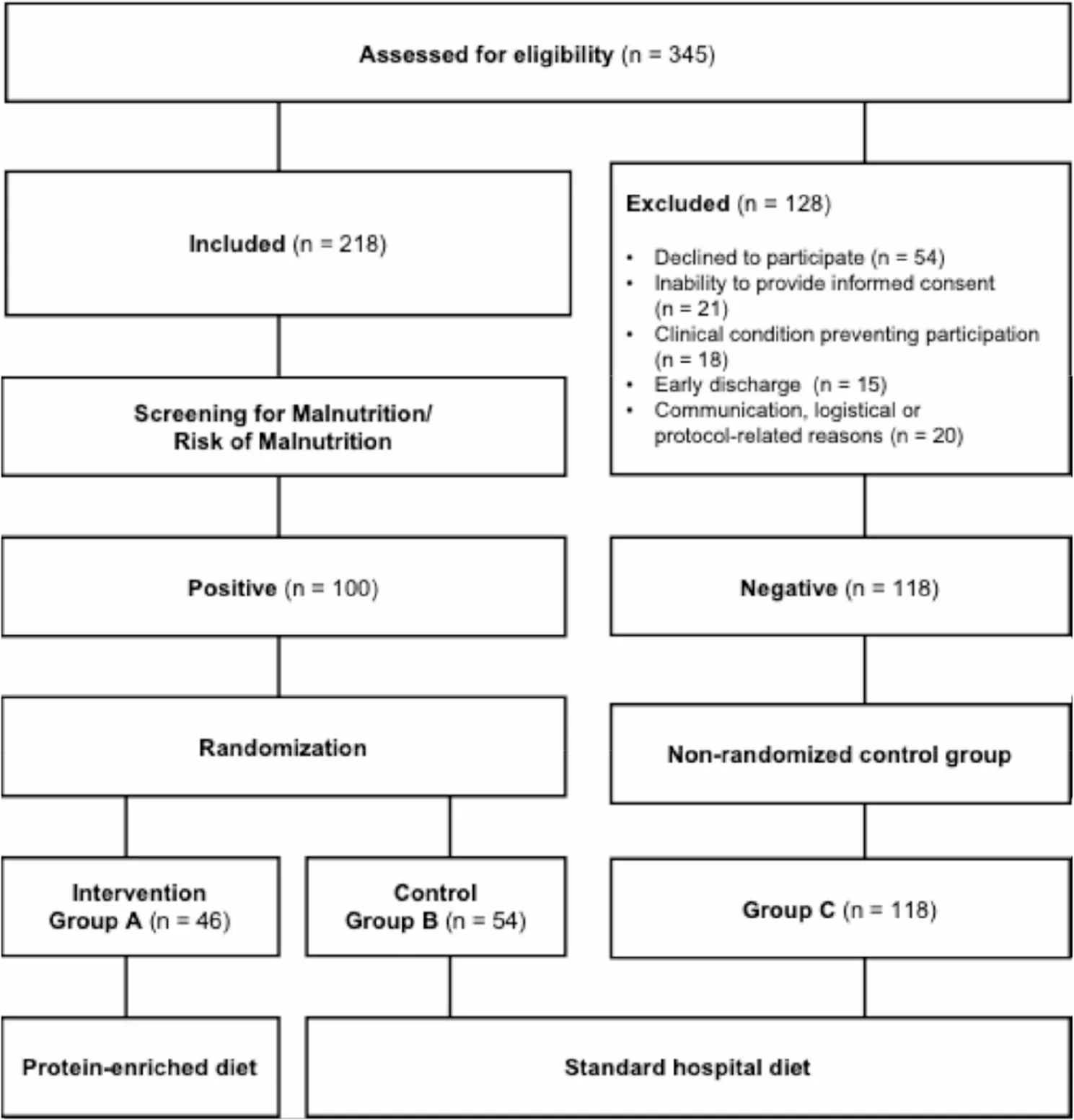
CONSORT-style flow diagram. Patients screened positive for being at nutritional risk were randomized to a protein-enriched diet (Group A) or standard hospital diet (Group B). Patients screened negative were included as a non-randomized observational reference group (Group C)

Nutritional risk was assessed within 48 h of admission using the Nutritional Risk Screening 2002 (NRS-2002) [27] and the full 18-item Mini Nutritional Assessment (MNA) [28]. An NRS-2002 score ≥ 3 indicated nutritional risk. MNA scores < 17 indicated malnutrition, scores of 17–23.5 indicated a risk of malnutrition, and scores ≥ 24 indicated normal nutritional status. Patients screened positive with at least one of the two instruments were assigned to the nutritional-risk group. Patients with negative results on both screenings were classified as not being at nutritional risk. As no confirmatory assessment according to the GLIM criteria was performed, this classification reflects nutritional risk based on screening and should not be interpreted as a diagnosis of malnutrition [29]. The screening algorithm is illustrated in Fig. 1.

Patients classified as being at nutritional risk were randomized to either the intervention group (Group A) or the nutritional-risk control group (Group B). Patients without nutritional risk formed a non-randomized observational reference group (Group C). In total, 218 patients were included in the analysis. Of these, 100 (45.9%) were classified as being at nutritional risk (Groups A and B), while 118 (54.1%) were classified as not being at nutritional risk (Group C).

The primary objective of the study was to test the hypothesis that a protein-enriched diet reduces post-traumatic complications and improves functional outcome in geriatric trauma patients at nutritional risk. Secondary objectives were to assess the impact of the intervention on length of hospital stay, discharge destination (return to home vs. community care vs. institutional care) and mortality.

### Randomization

Randomization was performed among geriatric trauma patients at nutritional risk. A computer-generated randomization sequence was created in Microsoft Excel for Microsoft 365 (Microsoft Corp., Redmond, WA, USA). Allocation to the nutritional-risk intervention group (A) or nutritional-risk control group (B) was performed on admission after informed consent and completion of nutritional screening to ensure unbiased group assignment.

### Nutritional intervention (Group A)

Patients in Group A received a structured protein-enriched nutritional regimen with a targeted total protein intake of 1.5–2.0 g/kg body weight per day, in accordance with current recommendations for older adults with acute illness [20]. This regimen combined three protein-rich main meals per day with oral nutritional supplements (ONS) providing 20 g protein per serving. The intervention was initiated as early as feasible after admission; the median time from admission to start of intervention was 1 day. Nutritional intake and tolerance were monitored as part of routine clinical care by the treating team and the clinical nutrition service; quantitative total energy and protein intake and adherence to ONS were not systematically recorded.

### Composition of protein-enriched diet

The protein-enriched diet, combined with ONS, was designed to achieve a total protein intake of approximately 2.0 g/kg body weight per day. No predefined caloric target was specified. The meals were standardized and consisted of three main meals and additional snacks incorporating protein-rich foods, such as dairy products (e.g. yoghurt, quark and cheese), eggs, meat, fish and plant-based proteins. Carbohydrate intake was primarily derived from complex carbohydrates (e.g. whole grains and potatoes), and fat intake included both saturated and unsaturated fatty acids. Energy-dense components were incorporated as clinically indicated. A detailed overview of nutrient composition is provided in the supplementary material.

### Standard care (Group B and C)

Patients in the nutritional-risk control group (B) and the reference group without nutritional risk (C) received standard hospital whole food diet without targeted protein enrichment or routine provision of oral nutritional supplements. Nutritional care followed local clinical practice and was not protocolized. Any additional nutritional measures outside the study protocol were recorded where applicable.

Apart from the nutritional intervention, concomitant care was provided according to routine clinical standards and did not differ by study allocation.

### Data collection and outcome measures

Baseline data included age, sex, fracture type, pre-injury living situation (independent, community care, institutional care), American Society of Anesthesiologists (ASA) classification and comorbidity burden according to the age-adjusted Charlson Comorbidity Index (ACCI).

### Geriatric assessment

All patients were treated in a geriatric trauma center including an orthogeriatric co-management. As part of routine care, standardized geriatric assessments were performed to evaluate vulnerability, frailty and delirium risk. These included the Identification of Seniors at Risk (ISAR), the Clinical Frailty Scale (CFS) and the Nursing Delirium Screening Scale (NuDESC). Screening results were extracted from the medical records for analysis.

As exploratory laboratory measures, serum albumin and C-reactive protein (CRP) concentrations were obtained at admission and discharge when available. Changes during hospitalization were analyzed only for patients with paired measurements of the respective biomarker.

### Primary outcomes

Primary outcomes were a priori defined as:

1. Medical complications during the index hospital stay, including:

- ⚬ Acute kidney injury (AKI), defined according to KDIGO criteria based on changes in serum creatinine levels (increase ≥ 0.3 mg/dL within 48 h or ≥ 1.5 times baseline within 7 days).
- ⚬ Urinary tract infection, based on clinical symptoms and laboratory parameters.
- ⚬ Pneumonia, based on clinical criteria and chest radiography.
- ⚬ Deep vein thrombosis, confirmed by duplex ultrasonography.

2. Surgical site infections in operatively treated patients, defined according to standard clinical and microbiological criteria.
3. Functional mobility pre-injury, at discharge (independent mobilization/ with assistive devices (e.g. walking frame, crutches)/ assisted standing/ bedbound) and individual changes.

### Secondary outcomes

1. Length of hospital stay (LOS) in days.
2. Discharge destination, categorized as: independently at home, community-based support or institutional care (e.g. nursing home, rehabilitation facility).
3. In-hospital mortality.

Intervention-related harms and tolerability concerns documented during routine clinical care were recorded; no structured adverse-event assessment was performed.

### Ethics and study regulations

The study was conducted in accordance with the Declaration of Helsinki. Ethical approval was obtained from the local ethics committee (758/2023BO2). All participants provided informed consent with the option to withdraw from the study at any time without the need for specification of reasons and without consequences for their medical treatment. The study was retrospectively registered at ClinicalTrials.gov on 12 May 2026 (NCT07582575). This manuscript was prepared in accordance with the CONSORT 2025 reporting guideline [30]. The completed CONSORT checklist is provided as supplementary material.

### Statistical analysis

Sample size calculations were performed using G*Power (Version 3). For the comparison of the randomized study arms (Group A and B), we assumed a clinically relevant effect size of 0.5 (Cohen’s d), a two-sided significance level of 0.05 and a power of 80%. This resulted in a required sample size of 64 patients per group. Accounting for an anticipated dropout rate of 20%, the target recruitment was set at 160 patients. The final analyzed sample comprised 46 patients in the intervention group and 54 patients in the control group.

Descriptive statistics were used to summarize baseline characteristics and outcomes. Continuous variables are presented as mean ± standard deviation. Depending on data distribution, group comparisons were performed using one-way ANOVA for normally distributed variables and the Kruskal–Wallis test for non-normally distributed variables. Categorical variables are presented as absolute and relative frequencies and were compared using a chi-squared test or an exact test when expected cell counts were small. Post hoc pairwise comparisons were adjusted using the Bonferroni correction.

To explore independent associations between patient characteristics, nutritional status and discharge destination, a multivariable multinomial logistic regression model was fitted, with independent discharge as the reference outcome. Covariates included sex, ACCI, fracture location (upper extremity vs. lower extremity/spine/pelvis) and study group. Group B, male sex, and lower extremity/spine/pelvic fractures were used as the respective reference categories. Age was not entered separately because it is incorporated into the ACCI. Adjusted odds ratios (aORs) with 95% confidence intervals (CI) are reported. Global effects of predictors were assessed using likelihood-ratio tests. Statistical significance was set at *p* ≤ 0.05. All analyses were conducted using IBM SPSS Statistics, version 29.0.2.0 (IBM Corp., Armonk, NY, USA).

## Results

### Baseline characteristics

A total of 218 patients were included in the analysis. The mean age was 81.8 ± 6.9 years, and 72.0% of patients were female (Table 1). Overall, 100 patients (45.9%) were classified as being at nutritional-risk and allocated to the intervention group (A; *n* = 46) or the control group (B; *n* = 54), while 118 patients (54.1%) formed the observational reference group without nutritional risk (C). Nearly half of all patients sustained a proximal femur fracture (48.6%), followed by pelvic fractures in 20.2% (Table 1). The majority of all patients received surgical treatment (*n* = 179, 82.1%) without between-group differences (*p* = 0.878). Patients at nutritional risk (Group A + B) had higher comorbidity burden according to ASA scores and ACCI scores than patients without nutritional risk (both *p* ≤ 0.003; Table 1).

**Table 1:** Baseline characteristics by study group (demographics, fracture types, comorbidity indices, LOS)

	Nutritional Risk		No nutritional Risk			
	Group A	Group B	Group C			
	Intervention	Control	Reference	Full cohort		
	*n* = 46	*n* = 54	*n* = 118	*n* = 218	**p-value**	**post-hoc**
Age [mean (± SD)]	83.7 (6.9)	82.4 (7.3)	80.8 (6.5)	81.8 (6.9)	0.054	n.s.
Sex [n (%)]						
Female	40 (87.0)	40 (74.1)	77 (65.3)	157 (72.0)	0.019	A vs. B *p* = 0.108
						B vs. C *p* = 0.250
Male	6 (13.0)	14 (25.9)	41 (34.7)	61 (28.0)		A vs. C *p* = 0.006
Fracture Type [n (%)]						
Proximal Humerus	3 (6.5)	4 (7.4)	23 (19.5)	30 (13.8)	0.171	
Vertebral Body	2 (4.3)	5 (9.3)	11 (9.3)	18 (8.3)		
Pelvic	10 (21.7)	13 (24.1)	21 (17.8)	44 (20.2)		n.s.
Proximal Femur	24 (52.2)	26 (48.1)	56 (47.5)	106 (48.6)		
Periprosthetic (LE)	7 (15.2)	6 (11.1)	7 (5.9)	20 (9.2)		
ASA Classification [n (%)]						
ASA I	4 (8.7)	5 (9.3)	14 (11.9)	23 (10.6)	0.003	A vs. B *p* = 0.581
ASA II	14 (30.4)	11 (20.4)	53 (44.9)	78 (35.8)		B vs. C *p* < 0.001
ASA III	26 (56.5)	33 (61.1)	51 (43.2)	110 (50.5)		A vs. C *p* = 0.039
ASA IV	2 (4.3)	5 (9.3)	0	7 (3.2)		
ACCI [mean (± SD)]	5.3 (1.3)	5.6 (2.0)	4.6 (1.5)	5.0 (1.7)	< 0.001	A vs. B *p* = 1.000
						B vs. C *p* = 0.002
						A vs. C *p* = 0.005
LOS [days, mean (± SD)]	11.5 (6.3)	11.6 (8.6)	10.2 (6.4)	10.8 (7.0)	0.367	

### Geriatric assessment

Geriatric assessment showed significantly higher ISAR and CFS scores in patients at nutritional risk (Group A + B) than in patients without nutritional risk, while delirium screening (NuDESC; *n* = 209 (A = 44; B = 44; C = 108)) did not differ between all groups (Table 2).

**Table 2:** Geriatric assessment

	Nutritional Risk		No nutritional Risk			
	Group A	Group B	Group C			
	Intervention	Control	Reference	Full cohort		
	*n* = 46	*n* = 54	*n* = 118	*n* = 218	**p-value**	**post-hoc**
					< 0.001	A vs. B *p* = 1.000
ISAR [median (IQR)]	2.0 (2.0)	2.0 (2.0)	1.0 (2.0)	2.0 (2.0)		B vs. C *p* < 0.001
						A vs. C *p* = 0.000
					< 0.001	A vs. B *p* = 1.000
CFS [median (IQR)]	5.0 (2.0)	4.0 (3.0)	3.0 (2.0)	3.0 (3.0)		B vs. C *p* < 0.001
						A vs. C *p* < 0.001
NuDESC [median (IQR)]	0.0 (0)	0 (0)	0.0 (0)	0.0 (0)	0.306	n.s.

### Laboratory trajectories during hospitalization

Exploratory analyses of paired laboratory measurements showed a mean change in Δ serum albumin of − 0.58 ± 0.44 g/dL among 122 patients, without significant differences among the groups (*p* = 0.777). Δ CRP baseline to discharge increased by a mean of 3.41 ± 5.77 mg/dL among 198 patients without significant between-group differences (*p* = 0.272).

### Medical complications and surgical site infections

At least one medical complication occurred in 98 of 218 patients (45.0%), without a significant difference among groups (*p* = 0.553). Acute kidney injury occurred in 21.1% of patients, urinary tract infection in 19.3%, pneumonia in 1.8%, and deep vein thrombosis in 2.8%; none differed significantly among groups (all *p* ≥ 0.163; Table 3).

**Table 3:** Complications

	Nutritional Risk		No nutritional Risk		
	Group A	Group B	Group C		
	Intervention	Control	Reference	Full cohort	
**Complication [n (%)]**	*n* = 46	*n* = 54	*n* = 118	*n* = 218	**p-value**
Any complication	18 (39.1)	27 (50.0)	53 (44.9)	98 (45.0)	0.553
Acute kidney injury	11 (23.9)	10 (18.5)	25 (21.2)	46 (21.1)	0.804
Urinary tract infection	5 (10.9)	14 (25.9)	23 (19.5)	42 (19.3)	0.163
Pneumonia	1 (2.2)	0	3 (2.5)	4 (1.8)	0.505
Deep vein thrombosis	1 (2.2)	3 (5.6)	2 (1.7)	6 (2.8)	0.344

Among the 179 operatively treated patients (82.1%), six surgical site infections occurred (3.4%) and differed significantly across the three study groups (Group A: 2.6%; Group B: 11.6%; Group C: 0.0%; *p* = 0.002). However, the difference between the randomized groups at nutritional risk was not statistically significant (Group A vs. Group B; *p* = 0.207).

### Functional mobility

Pre-injury mobility differed significantly among the groups (*p* < 0.001). Patients in Group C were more frequently mobile without assistive devices before the fracture (79.7%) than patients in Groups A (45.7%) and B (50.0%; Table 4). Mobility at discharge also differed significantly among the groups (*p* < 0.001) with patients in Group C more frequently using crutches, whereas patients in Groups A and B more frequently required a walker (Table 4). Post-hoc comparisons showed significant differences between Groups A and C (*p* < 0.001) and between Groups B and C (*p* = 0.005), but not between Groups A and B (*p* = 0.571; Table 4). However, deterioration from pre-injury mobility to mobility at discharge occurred in almost the entire cohort and did not differ significantly among the groups (Group A: 100.0%; Group B: 98.1%; Group C: 98.3%; *p* = 1.000) (Table 4). The best post-traumatic mobility differed significantly between the groups; patients without nutritional risk were significantly more likely to be mobile without assistive devices (Table 4). The intervention had no effect on this (*p* = 0.541).

**Table 4:** Mobility pre- and post-injury according to the use of assistive devices, change in mobility status and best ambulation

	Nutritional Risk		No nutritional Risk			
	Group A	Group B	Group C			
	Intervention	Control	Reference	Full cohort		
	*n* = 46	*n* = 54	*n* = 118	*n* = 218	**p-value**	**post-hoc**
Pre-injury mobility / Use of assistive devices [n (%)]						
None	21 (45.7)	27 (50.0)	94 (79.7)	142 (65.1)	< 0.001	A vs. B *p* = 1.00 B vs. C = *p* < 0.001 A vs. C = *p* < 0.001
Crutches	10 (21.7)	5 (9.3)	11 (9.3)	26 (11.9)		
Walker	15 (32.6)	20 (37.0)	12 (10.2)	47 (21.6)		
Wheelchair	0 (0.0)	1 (1.9)	1 (0.8)	2 (0.9)		
Bedridden	0 (0.0)	1 (1.9)	0 (0.0)	1 (0.5)		
Post-injury mobility / Use of assistive devices [n (%)]						
None	0 (0.0)	0 (0.0)	0 (0.0)	0 (0.0)	< 0.001	A vs. B *p* = 0.571 B vs. C *p* = 0.005 A vs. C *p* < 0.001
Crutches	6 (13.04)	15 (27.78)	62 (52.54)	83 (38.07)		
Walker	36 (78.26)	34 (62.96)	51 (43.22)	121 (55.50)		
Wheelchair	2 (4.35)	2 (3.70)	4 (3.39)	8 (3.67)		
Bedridden	2 (4.35)	3 (5.56)	1 (0.85)	6 (2.80)		
Change in mobility status [n (%)]						
None	0 (0.0)	0 (0.0)	0 (0.0)	0 (0.0)	1.000	
Improvement	0 (0.0)	1 (1.9)	2 (1.7)	3 (1.4)		n.s.
Deterioration	46 (100.0)	53 (98.1)	116 (98.3)	215 (98.6)		
Best ambulation [n (%)]						
Independent ambulation	1 (2.2)	7 (13.0)	27 (22.9)	35 (16.1)	0.014	A vs. B *p* = 0.541 B vs. C *p* = 0.066 A vs. C *p* = 0.001
Ambulation with assistive devices	38 (82.6)	40 (74.1)	86 (72.9)	164 (75.2)		
Assisted standing	5 (10.9)	5 (9.3)	4 (3.4)	14 (6.4)		
Bedbound	2 (4.3)	2 (3.7)	1 (0.8)	5 (2.3)		

### Length of hospital stay and discharge destination

The length of hospital stay was similar across groups (*p* = 0.367; Table 5). Pre-injury, two thirds of all patients (66.5%) lived independently. Patients at nutritional risk showed significantly higher rates of dependent living prior to injury compared to patients without nutritional risk (Table 5). At discharge, only 24.8% of all patients returned home without additional support, 16.5% required community care, and 58.7% were discharged to institutional care (Table 5). Discharge destination differed significantly among the groups (*p* = 0.001). In post hoc comparisons, independent living at discharge was more frequent in group C than in Group A (*p* = 0.001), whereas no significant differences were observed between the other groups (Table 5).

**Table 5:** Pre-injury living situation, discharge destination, and changes in care requirements by study group

	Nutritional Risk		No nutritional Risk			
	Group A	Group B	Group C			
	Intervention	Control	Reference	Full cohort		
	*n* = 46	*n* = 54	*n* = 118	*n* = 218	**p-value**	**post-hoc**
Pre-injury living situation [n (%)]						
Independent	22 (47.8)	29 (53.7)	94 (79.7)	145 (66.5)	< 0.001	A vs. B *p* = 1.000
Community care	21 (45.7)	15 (27.8)	20 (16.9)	56 (25.7)		B vs. C *p* = 0.001
Institutional care	3 (6.5)	10 (18.5)	4 (3.4)	17 (7.8)		A vs. C *p* < 0.001
Discharge destination [n (%)]						
Independent	6 (13.0)	12 (22.2)	36 (30.5)	54 (24.8)	0.001	A vs. B *p* = 0.295
Community care	3 (6.6)	8 (14.8)	25 (21.2)	36 (16.5)		B vs. C *p* = 0.255
Institutional care	37 (80.4)	34 (63.0)	57 (48.3)	128 (58.7)		A vs. C *p* = 0.001
Change in living situation [n (%)]						
No deterioration	12 (26.1)	25 (46.3)	47 (39.8)	84 (38.5)	0.107	n.s.
Deterioration	34 (73.9)	29 (53.7)	71 (60.2)	134 (61.5)		

In an exploratory analysis comparing pre-injury and discharge living situations, an increased level of care was observed in 73.9% of patients in Group A, 53.7% in Group B, and 60.2% in Group C. These proportions did not differ significantly among the groups (*p* = 0.107). In a post hoc subgroup analysis restricted to patients who had lived independently before the fracture (*n* = 145), loss of independent living at discharge occurred in 72.7% of patients in Group A, 58.6% in Group B, and 61.7% in Group C, without a significant between-group difference (*p* = 0.551).

No patient died during the index hospitalization.

In the multivariable multinomial logistic regression with independent discharge as the reference outcome (Table 6), a higher ACCI was independently associated with discharge to community support (aOR 1.73, 95% CI 1.24–2.42; *p* = 0.001) and institutional care (aOR 1.75, 95% CI 1.31–2.34; *p* < 0.001). Study group showed a significant overall association with discharge destination (*p* = 0.025). However, compared with patients at nutritional risk receiving the standard hospital diet (Group B), patients receiving the protein-enriched diet with ONS (Group A) did not have significantly different odds of discharge to community support (aOR 0.71, 95% CI 0.13–3.83; *p* = 0.690) or institutional care (aOR 2.25, 95% CI 0.72–7.05; *p* = 0.163). Upper-extremity fractures were associated with lower odds of institutional care compared with independent discharge (aOR 0.32, 95% CI 0.12–0.85; *p* = 0.022), although the overall association of fracture location with discharge destination did not reach statistical significance (*p* = 0.068). Sex was not independently associated with discharge destination (*p* = 0.175; Table 6). The overall model was statistically significant ( *p* < 0.001).

**Table 6:** Multivariable multinomial logistic regression predicting discharge destination. Adjusted odds ratios (aORs) with 95% confidence intervals (CIs) comparing community care and institutional care versus independent discharge. Global p-values were derived from likelihood-ratio tests. The model included sex, ACCI, fracture location (upper extremity vs. lower extremity/spine/pelvis), and study group (Group A = patients at nutritional risk receiving a protein-enriched diet with ONS; Group B = patients at nutritional risk receiving the standard hospital diet; Group C = patients without nutritional risk)

Predictor	Global *p*-value	Community care vs. independent discharge, aOR (95% CI)	*p*-value	Institutional care vs. independent discharge, aOR (95% CI)	*p*-value
Female vs. male	0.175	2.59 (0.90–7.44)	0.078	1.27 (0.59–2.74)	0.534
ACCI, per one-point increase	< 0.001	1.73 (1.24–2.42)	0.001	1.75 (1.31–2.34)	< 0.001
Fractures of the upper-extremity vs. lower extremity/spine/pelvis	0.068	0.57 (0.18–1.78)	0.335	0.32 (0.12–0.85)	0.022
Study group	0.025				
Group A vs. Group B		0.71 (0.13–3.83)	0.690	2.25 (0.72–7.05)	0.163
Group C vs. Group B		1.83 (0.60–5.62)	0.290	0.99 (0.42–2.35)	0.978

No intervention-related harms or unintended events were observed in any group during the trial.

## Discussion

In this prospective geriatric trauma cohort, almost half of the patients screened positive for nutritional risk at admission. Nutritional risk was associated with greater frailty, comorbidity, and pre-injury care requirements. However, among patients at nutritional risk, the protein-enriched diet did not significantly reduce medical complications or surgical site infections and did not improve functional mobility, length of hospital stay, or discharge destination compared with the standard hospital diet.

### Cohort characteristics and nutritional risk

Consistent with the epidemiology of fragility fractures, the cohort was predominantly female (72.0%). Several major fragility-fracture locations were included, reflecting a broader inpatient geriatric trauma population than cohorts restricted to proximal femur fractures. Lower-extremity fractures nevertheless predominated, whereas vertebral and proximal humeral fractures were less common. This distribution may reflect the frequent non-operative and outpatient management of vertebral and proximal humeral fractures, which reduces the number of such patients available for recruitment during hospitalization. The findings therefore apply primarily to hospitalized older adults with fragility fractures and may be less generalizable to patients managed entirely in outpatient settings.

Our findings are consistent with previous research showing that malnutrition in geriatric trauma patients is strongly linked to frailty, multimorbidity and adverse clinical outcomes, including complications and mortality [10–12, 14, 17]. In hip fracture cohorts, patients with malnutrition or at risk of malnutrition show disability in activities of daily living according to the Barthel Index before and up to six months after fracture [18]. In our study, patients at nutritional risk had higher ISAR and CFS scores, higher ASA classes and greater comorbidity according to the ACCI than the reference group without nutritional risk. This supports the idea, that being at nutritional risk in geriatric trauma patients is not an isolated entity but part of the geriatric syndrome [1, 3].

### Serum albumin and CRP

Low serum albumin has repeatedly been associated with postoperative complications and mortality in orthopedic surgery, including among patients undergoing arthroplasty after hip fracture [14, 17, 19]. Preoperative hypoalbuminemia has also been associated with reoperation in this population [19]. We examined changes in serum albumin and CRP to explore whether the protein-enriched diet was accompanied by measurable short-term biological changes during hospitalization. Serum albumin decreased in all groups, without a significant difference in its trajectory among groups. During the acute post-fracture phase, systemic inflammation, surgical trauma, and fluid shifts strongly affect albumin homeostasis. These short-term changes are unlikely to be reversed by nutritional measures during a single hospital stay, and current evidence does not support serum albumin as a reliable marker of response to nutritional therapy [17, 31–33]. CRP increased from admission to discharge, again without a significant difference among groups. Thus, the protein-enriched diet was not accompanied by a measurably different short-term inflammatory trajectory. However, the absence of between-group differences in albumin or CRP should not be interpreted as evidence against a biological effect of nutritional supplementation, as both biomarkers are strongly influenced by the acute inflammatory response, while CRP may additionally reflect trauma-related tissue injury rather than a specific response to nutritional treatment [34].

### Medical complications

Within the nutritional-risk subgroup, the intervention did not significantly reduce medical complications or surgical site infections compared with the standard hospital diet. In very old and multimorbid patients, comorbid disease, pre-fracture disability, and social circumstances may have a stronger influence on short-term in-hospital outcomes than an acute increase in protein provision [21, 26]. Although the difference did not reach statistical significance, the numerically lower rate of surgical site infections in the intervention group warrants further investigation in adequately powered trials, particularly in light of previous evidence suggesting that ONS may reduce infectious complications after hip fracture surgery [22, 35].

### Functional mobility and length of hospital stay

Beyond its potential effects on complications, nutritional status may influence functional recovery. In our cohort, patients at nutritional risk had less favorable functional mobility at discharge than those without nutritional risk. However, the patients at nutritional risk also had poorer pre-injury mobility, greater frailty, and greater comorbidity burdens. The intervention did not improve functional mobility at discharge, suggesting that the short-term protein-enriched diet alone was insufficient to produce a measurable early functional benefit.

The intervention also did not shorten the length of hospital stay. Although some earlier studies have suggested that ONS may modestly reduce length of stay in selected populations [21–25], we did not observe an influence of nutritional status or intervention on length of hospital stay. This finding must be interpreted in the context of the Diagnosis-related-Groups (DRG)-based reimbursement system in the study country, where lengths of hospital stay for geriatric trauma patients are frequently standardized around a narrow time window, commonly around postoperative days 11–12 [36]. Because length of stay is largely standardized by the DRG system, there is little room for variation. Any small effect of nutritional status on discharge timing is therefore difficult to detect. Hence, our results do not exclude potential benefits of improved nutrition on outcomes beyond the acute hospitalization, such as rehabilitation duration, readmissions or long-term institutionalization, which were not assessed in this analysis.

### Discharge destination and living situation

Patients at nutritional risk were more likely to receive community-based or institutional care before the fracture than patients without nutritional risk. However, when changes in living arrangements were assessed relative to the pre-injury situation, no significant differences were observed among the groups. Moreover, the protein-enriched diet with ONS did not increase the likelihood of returning home. In multivariable analysis, a higher ACCI was independently associated with discharge to community support or institutional care rather than independent discharge. No independent effect of the nutritional intervention was observed. This suggests that being at nutritional risk in this context primarily acts as a marker of overall vulnerability. Baseline function, pre-injury living situation and the overall burden of chronic disease seem to be more important for early discharge outcomes than nutritional status alone [1, 26].

### Comparison with previous trials on oral nutritional supplementation

Optimizing protein intake in geriatric, multimorbid patients is considered important for muscle maintenance and long-term frailty prevention, but the expected short-term effects during a single hospital stay are modest [10, 37, 38]. Large randomized trials in hospitalized and critically ill adults have not shown improvements in mortality or quality of life with higher protein doses and have even raised safety concerns in selected subgroups, underscoring the need for individualized strategy [39, 40]. In geriatric hip fracture patients, ONS may reduce overall complication rates, pressure ulcer incidence and may improve functional recovery in selected settings, while effects on surgical site infections, length of hospital stay and mortality are less consistent [21–25, 41]. In contrast, we did not observe differences in medical complication rates, surgical site infections or in-hospital mortality between patients at nutritional risk receiving the protein-enriched diet and those on standard hospital diet.

Several features of our study may explain this discrepancy. Firstly, the majority of previous trials focused exclusively on hip fractures, whereas the present cohort included a broader spectrum of fragility fractures and proximal femur fractures accounted for approximately half of all cases [21, 22]. Secondly, our intervention was restricted to the acute hospital stay. The absence of measurable effects may therefore partly reflect the short intervention period. Studies examining protein-related gains in muscle mass and strength have generally used interventions lasting several weeks to months, often combined with resistance training; although these settings are not directly transferable to acute orthogeriatric care, they illustrate the longer time frame commonly required for measurable functional adaptation [42, 43]. Future studies should therefore evaluate prolonged protein supplementation combined with physical exercise during post-fracture rehabilitation.

### Clinical implications and future directions

Overall, our data suggest that being at nutritional risk in geriatric trauma patients can serve as a marker of advanced frailty and multimorbidity rather than a short-term modifiable risk factor [1, 3, 10, 11]. Identification of nutritional risk should therefore trigger comprehensive orthogeriatric assessment and multimodal interventions, including early mobilization, fall prevention, medication review and structured discharge planning, rather than being addressed in isolation [9, 13].

A protein-enriched diet during acute hospital stay is feasible and consistent with current recommendations for geriatric patients [10, 11, 17, 20], but our results suggest that protein supplementation alone is unlikely to substantially change early in-hospital outcomes in geriatric trauma patients.

Future studies should therefore evaluate integrated strategies that combine appropriate energy and protein intake with resistance exercise, early and intensive mobilization and rehabilitation-focused care pathways. Objective measures of sarcopenia, systematic assessment of adherence to supplementation and the use of digital tools (e.g. wearable sensors) for continuous monitoring of mobility could help to identify responders and define the time frame over which nutritional interventions exert clinically meaningful effects. Longitudinal follow-up including activities of daily living, quality of life, readmissions and long-term living situation will be crucial to fully assess the impact of nutritional strategies in orthogeriatric trauma care.

### Limitations

These findings should be interpreted in light of several limitations. The study was conducted at a single academic Level I trauma center within a specific orthogeriatric care model and the German diagnosis-related group reimbursement framework. Although this provided a well-defined care context, it limits generalizability to other populations, institutions, and healthcare systems. Multicenter studies are needed to assess the external validity of these findings.

The randomized groups were smaller than planned: the target of 64 evaluable patients per group was not reached, with 46 patients analyzed in Group A and 54 in Group B. The trial was therefore underpowered to detect the prespecified effect and especially to assess infrequent outcomes such as surgical site infection. The non-significant randomized comparisons cannot exclude a clinically relevant intervention effect and should be interpreted cautiously. In addition, comparisons with the non-randomized Group C are observational and remain susceptible to residual confounding despite multivariable adjustment. Regarding the multivariable multinomial logistic regression model, pre-injury living situation was initially considered as an additional covariate. However, its inclusion resulted in separation and unstable parameter estimates; it was therefore not retained in the final model. Consequently, residual confounding by pre-injury living situation cannot be excluded.

The intervention was confined to the acute hospital stay. Total energy and protein intake and adherence to ONS were not systematically quantified; previous work has shown substantial variation in intake and that poor adherence may attenuate the effectiveness of supplementation [22, 23, 25].

Consequently, achievement of the intended protein target cannot be confirmed, and displacement of regular food intake by ONS cannot be excluded. Body weight and nutritional screening scores were not systematically reassessed at discharge; changes in nutritional status during hospitalization therefore could not be determined. Post-discharge nutrition and rehabilitation pathways were also not systematically recorded.

Only 28% of the study population was male, including six males in the intervention group. Although this distribution is broadly consistent with the epidemiology of fragility fractures and sex was included in the multinomial model, the number of male participants was insufficient for adequately powered sex-stratified analyses or assessment of sex-specific intervention effects.

Furthermore, the inclusion of different fracture types with varying treatment pathways introduced clinical heterogeneity. At the same time, the inclusion of a broad spectrum of fragility fractures represents a strength of this study, as previous nutritional intervention studies have predominantly focused on patients with proximal femur fractures. Our study therefore extends the investigation of nutritional interventions to a more diverse and clinically representative orthogeriatric population. Nevertheless, although fracture location was considered in the adjusted analysis, fracture-specific intervention effects could not be assessed because of the limited subgroup sizes.

Finally, although 120-day and 12-month follow-up will be available for a smaller subgroup, the present analysis focuses on in-hospital outcomes, and long-term effects of nutritional status and supplementation on function and living situation in the full cohort remain to be determined.

## Supplementary Information

Supplementary Material 1.

## Data Availability

The datasets generated and/or analyzed during the current study are available from the corresponding author on reasonable request.

## Abbreviations

ASA: American Society of Anesthesiologists physical status classification
ACCI: Age-adjusted Charlson Comorbidity Index
CFS: Clinical Frailty Scale
ISAR: Identification of Seniors at Risk
LOS: Length of hospital stay
MNA: Mini Nutritional Assessment
NRS: Nutritional Risk Screening
NuDESC: Nursing Delirium Screening Scale
NuTra: Nutrition and Trauma trial
ONS: Oral Nutritional Supplements
aOR: Adjusted Odds ratio
SD: Standard deviation

## References

[1] Clegg A, Young J, Iliffe S, Rikkert MO, Rockwood K. Frailty in Elderly People. Lancet. 2013;381:752–62. 10.1016/S0140-6736(12)62167-9. PMC4098658 23395245

[2] Borgström F, Karlsson L, Ortsäter G, Norton N, Halbout P, Cooper C, Lorentzon M, McCloskey EV, Harvey NC, Javaid MK, et al. Fragility Fractures in Europe: Burden, Management and Opportunities. Arch Osteoporos. 2020;15:59. 10.1007/s11657-020-0706-y. PMC7166207 32306163

[3] Inoue T, Maeda K, Nagano A, Shimizu A, Ueshima J, Murotani K, Sato K, Tsubaki A, Undernutrition. Sarcopenia, and Frailty in Fragility Hip Fracture: Advanced Strategies for Improving Clinical Outcomes. Nutrients. 2020;12:3743. 10.3390/nu12123743. PMC7762043 33291800

[4] Migliorini F, Giorgino R, Hildebrand F, Spiezia F, Peretti GM, Alessandri-Bonetti M, Eschweiler J, Maffulli N. Fragility Fractures: Risk Factors and Management in the Elderly. Medicina. 2021;57:1119. 10.3390/medicina57101119. PMC8538459 34684156

[5] Rupp M, Walter N, Pfeifer C, Lang S, Kerschbaum M, Krutsch W, Baumann F, Alt V. The Incidence of Fractures Among the Adult Population of Germany–an Analysis From 2009 through 2019. Dtsch Arztebl Int. 2021;118:665–9. 10.3238/arztebl.m2021.0238. PMC8727861 34140088

[6] Bleibler F, Konnopka A, Benzinger P, Rapp K, König H-H. The Health Burden and Costs of Incident Fractures Attributable to Osteoporosis from 2010 to 2050 in Germany—a Demographic Simulation Model. Osteoporos Int. 2013;24:835–47. 10.1007/s00198-012-2020-z. 22797490

[7] Veronese N, Maggi S. Epidemiology and Social Costs of Hip Fracture. Injury. 2018;49:1458–60. 10.1016/j.injury.2018.04.015. 29699731

[8] Ruiz-Adame M, Correa MA. Systematic Review of the Indirect and Social Costs Studies in Fragility Fractures. Osteoporos Int. 2020;31:1205–16. 10.1007/s00198-020-05319-x. 32002572

[9] Pollmann CT, Mellingsæter MR, Neerland BE, Straume-Næsheim T, Årøen A, Watne LO. Orthogeriatric Co-Management Reduces Incidence of Delirium in Hip Fracture Patients. Osteoporos Int. 2021;32:2225–33. 10.1007/s00198-021-05974-8. PMC8563591 33963884

[10] Cruz-Jentoft AJ, Volkert D. Malnutrition in Older Adults. N Engl J Med. 2025;392:2244–55. 10.1056/NEJMra2412275. 40499173

[11] Dent E, Wright ORL, Woo J, Hoogendijk EO. Malnutrition in Older Adults. Lancet. 2023;401:951–66. 10.1016/S0140-6736(22)02612-5. 36716756

[12] Meermans G, Egmond J.C. van, Meermans G, Egmond J.C. van Malnutrition in Older Hip Fracture Patients: Prevalence, Pathophysiology, Clinical Outcomes, and Treatment—A Systematic Review. J Clin Med. 2025;14. 10.3390/jcm14165662. PMC12386537 40869488

[13] Liener UC, Becker C, Rapp K, Raschke MJ, Kladny B, Wirtz DC. Deutsche Gesellschaft für Orthopädie und Unfallchirurgie, Deutsche Gesellschaft für Geriatrie, Eds.; 1. Auflage.; Verlag W. Kohlhammer: Stuttgart, 2022; ISBN 978-3-17-041174-6.

[14] Foo MXE, Wong GJY, Lew CCH. A Systematic Review of the Malnutrition Prevalence in Hospitalized Hip Fracture Patients and Its Associated Outcomes. J Parenter Enter Nutr. 2021;45:1141–52. 10.1002/jpen.2211. 34169533

[15] Cederholm T, Bosaeus I. Malnutrition in Adults. N Engl J Med. 2024;391:155–65. 10.1056/NEJMra2212159. 38986059

[16] Ligthart-Melis GC, Luiking YC, Kakourou A, Cederholm T, Maier AB, Schueren MAE, de van der Frailty S, Frequently M. (Co-)Occur in Hospitalized Older Adults: A Systematic Review and Meta-Analysis. J Am Med Dir Assoc. 2020;21:1216–28. 10.1016/j.jamda.2020.03.006. 32327302

[17] Schuetz P, Seres D, Lobo DN, Gomes F, Kaegi-Braun N, Stanga Z. Management of Disease-Related Malnutrition for Patients Being Treated in Hospital. Lancet. 2021;398:1927–38. 10.1016/S0140-6736(21)01451-3. 34656286

[18] Goisser S, Schrader E, Singler K, Bertsch T, Gefeller O, Biber R, Bail HJ, Sieber CC, Volkert D. Malnutrition According to Mini Nutritional Assessment Is Associated With Severe Functional Impairment in Geriatric Patients Before and up to 6 Months After Hip Fracture. J Am Med Dir Assoc. 2015;16:661–7. 10.1016/j.jamda.2015.03.002. 25864084

[19] Newman JM, Sodhi N, Khlopas A, Piuzzi NS, Yakubek GA, Sultan AA, Klika AK, Higuera CA, Mont MA. Malnutrition Increases the 30-Day Complication and Re-Operation Rates in Hip Fracture Patients Treated with Total Hip Arthroplasty. HIP Int. 2020;30:635–40. 10.1177/1120700019862977. 31304789

[20] Volkert D, Beck AM, Cederholm T, Cruz-Jentoft A, Hooper L, Kiesswetter E, Maggio M, Raynaud-Simon A, Sieber C, Sobotka L, et al. ESPEN Practical Guideline: Clinical Nutrition and Hydration in Geriatrics. Clin Nutr. 2022;41:958–89. 10.1016/j.clnu.2022.01.024. 35306388

[21] Avenell A, Smith TO, Curtain JP, Mak JC, Myint PK. Nutritional Supplementation for Hip Fracture Aftercare in Older People. Cochrane Database Syst Reviews. 2016. 10.1002/14651858.CD001880.pub6. PMC6464805 27898998

[22] Chen B, Zhang JH, Duckworth AD, Clement ND. Effect of Oral Nutritional Supplementation on Outcomes in Older Adults with Hip Fractures and Factors Influencing Compliance: A Systematic Review and Meta-Analysis of Randomized Controlled Trials. Bone Joint J. 2023;105–B:1149–58. 10.1302/0301-620X.105B11.BJJ-2023-0139.R1. 37907073

[23] Espaulella J, Guyer H, Diaz-Escriu F, Mellado-Navas JA, Castells M, Pladevall M. Nutritional Supplementation of Elderly Hip Fracture Patients. A Randomized, Double-Blind, Placebo-Controlled Trial. Age Ageing. 2000;29:425–31. 10.1093/ageing/29.5.425. 11108415

[24] Tidermark J, Ponzer S, Carlsson P, Söderqvist A, Brismar K, Tengstrand B, Cederholm T. Effects of Protein-Rich Supplementation and Nandrolone in Lean Elderly Women with Femoral Neck Fractures. Clin Nutr. 2004;23:587–96. 10.1016/j.clnu.2003.10.006. 15297095

[25] Myint MWW, Wu J, Wong E, Chan SP, To TSJ, Chau MWR, Ting KH, Fung PM, Au KSD. Clinical Benefits of Oral Nutritional Supplementation for Elderly Hip Fracture Patients: A Single Blind Randomised Controlled Trial. Age Ageing. 2013;42:39–45. 10.1093/ageing/afs078. 22685164

[26] Han TS, Yeong K, Lisk R, Fluck D, Fry CH. Prevalence and Consequences of Malnutrition and Malnourishment in Older Individuals Admitted to Hospital with a Hip Fracture. Eur J Clin Nutr. 2021;75:645–52. 10.1038/s41430-020-00774-5. 33028971

[27] Kondrup J, Rasmussen HH, Hamberg O, Stanga Z, Ad Hoc ESPEN, Working Group Nutritional Risk Screening (NRS 2002). A New Method Based on an Analysis of Controlled Clinical Trials. Clin Nutr. 2003;22:321–36. 10.1016/s0261-5614(02)00214-5. 12765673

[28] Guigoz, Y. The Mini Nutritional Assessment (MNA) Review of the Literature-What Does It Tell Us? J Nutr Health Aging 2006, 10, 466–485; discussion 485-487. 17183419

[29] Cederholm T, Jensen GL, Correia MITD, Gonzalez MC, Fukushima R, Higashiguchi T, Baptista G, Barazzoni R, Blaauw R, Coats AJS, et al. GLIM Criteria for the Diagnosis of Malnutrition – A Consensus Report from the Global Clinical Nutrition Community. J Cachexia Sarcopenia Muscle. 2019;10:207–17. 10.1002/jcsm.12383. PMC6438340 30920778

[30] Hopewell S, Chan A-W, Collins GS, Hróbjartsson A, Moher D, Schulz KF, Tunn R, Aggarwal R, Berkwits M, Berlin JA, et al. CONSORT 2025 Statement: Updated Guideline for Reporting Randomised Trials. BMJ. 2025;389:e081123. 10.1136/bmj-2024-081123. PMC11995449 40228833

[31] Eckart A, Struja T, Kutz A, Baumgartner A, Baumgartner T, Zurfluh S, Neeser O, Huber A, Stanga Z, Mueller B, et al. Relationship of Nutritional Status, Inflammation, and Serum Albumin Levels During Acute Illness: A Prospective Study. Am J Med. 2020;133:713–e7227. 10.1016/j.amjmed.2019.10.031. 31751531

[32] Evans DC, Corkins MR, Malone A, Miller S, Mogensen KM, Guenter P, Jensen GL. Committee, the A.M. The Use of Visceral Proteins as Nutrition Markers: An ASPEN Position Paper. Nutr Clin Pract. 2021;36:22–8. 10.1002/ncp.10588. 33125793

[33] Boesiger F, Poggioli A, Netzhammer C, Bretscher C, Kaegi-Braun N, Tribolet P, Wunderle C, Kutz A, Lobo DN, Stanga Z, et al. Changes in Serum Albumin Concentrations over 7 Days in Medical Inpatients with and without Nutritional Support. A Secondary Post-Hoc Analysis of a Randomized Clinical Trial. Eur J Clin Nutr. 2023;77:989–97. 10.1038/s41430-023-01303-w. PMC10564620 37419969

[34] Zhou H-H, Tang Y-L, Xu T-H, Cheng BC-R, Protein. Structure, Function, Regulation, and Role in Clinical Diseases. Front Immunol. 2024;15:1425168. 10.3389/fimmu.2024.1425168. PMC11211361 38947332

[35] He Y, Xiao J, Shi Z, He J, Li T. Supplementation of Enteral Nutritional Powder Decreases Surgical Site Infection, Prosthetic Joint Infection, and Readmission after Hip Arthroplasty in Geriatric Femoral Neck Fracture with Hypoalbuminemia. J Orthop Surg Res. 2019;14:292. 10.1186/s13018-019-1343-2. PMC6724262 31481078

[36] Busse R, Blümel M, Knieps F, Bärnighausen T. Statutory Health Insurance in Germany: A Health System Shaped by 135 Years of Solidarity, Self-Governance, and Competition. Lancet. 2017;390:882–97. 10.1016/S0140-6736(17)31280-1. 28684025

[37] Mendonça N, Kingston A, Granic A, Jagger C. Protein Intake and Transitions between Frailty States and to Death in Very Old Adults: The Newcastle 85 + Study. Age Ageing. 2020;49:32–8. 10.1093/ageing/afz142. PMC6939283 31711099

[38] Harris S, DePalma J, Barkoukis H, Harris S, DePalma J, Barkoukis H. Protein and Aging: Practicalities and Practice. Nutrients. 2025;17. 10.3390/nu17152461. PMC12348035 40806046

[39] Heyland DK, Patel J, Compher C, Rice TW, Bear DE, Lee Z-Y, González VC, O’Reilly K, Regala R, Wedemire C, et al. The Effect of Higher Protein Dosing in Critically Ill Patients with High Nutritional Risk (EFFORT Protein): An International, Multicentre, Pragmatic, Registry-Based Randomised Trial. Lancet. 2023;401:568–76. 10.1016/S0140-6736(22)02469-2. 36708732

[40] Bels JLM, Thiessen S, van Gassel RJJ, Beishuizen A, De Bie Dekker A, Fraipont V, Lamote S, Ledoux D, Scheeren C, De Waele E, et al. Effect of High versus Standard Protein Provision on Functional Recovery in People with Critical Illness (PRECISe): An Investigator-Initiated, Double-Blinded, Multicentre, Parallel-Group, Randomised Controlled Trial in Belgium and the Netherlands. Lancet. 2024;404:659–69. 10.1016/S0140-6736(24)01304-7. 39153816

[41] Lai W-Y, Chiu Y-C, Lu K-C, Huang I-T, Tsai P-S, Huang C-J. Beneficial Effects of Preoperative Oral Nutrition Supplements on Postoperative Outcomes in Geriatric Hip Fracture Patients: A PRISMA-Compliant Systematic Review and Meta-Analysis of Randomized Controlled Studies. Medicine. 2021;100:e27755. 10.1097/MD.0000000000027755. PMC8615416 34964732

[42] Morton RW, Murphy KT, McKellar SR, Schoenfeld BJ, Henselmans M, Helms E, Aragon AA, Devries MC, Banfield L, Krieger JW, et al. A Systematic Review, Meta-Analysis and Meta-Regression of the Effect of Protein Supplementation on Resistance Training-Induced Gains in Muscle Mass and Strength in Healthy Adults. Br J Sports Med. 2018;52:376–84. 10.1136/bjsports-2017-097608. PMC5867436 28698222

[43] Cereda E, Pisati R, Rondanelli M, Caccialanza RW, Protein. Leucine- and Vitamin-D-Enriched Oral Nutritional Supplementation for the Treatment of Sarcopenia. Nutrients. 2022;14:1524. 10.3390/nu14071524. PMC9003251 35406137

